# Telehealth utilization in U.S. Medicare beneficiaries aged 65 years and older during the COVID-19 pandemic

**DOI:** 10.1186/s12889-023-15263-0

**Published:** 2023-02-20

**Authors:** Min Lu, Xinyi Liao

**Affiliations:** Department of Public Health Sciences, Miller School of Medicine, University of Miami, Miami, Florida, USA

**Keywords:** COVID-19, Older adults, Medicare, Primary care, Telehealth, Telemedicine

## Abstract

**Background:**

The COVID-19 pandemic has become a serious public health concern for older adults and amplified the value of deploying telehealth solutions. The purpose of this study was to investigate telehealth offered by providers among U.S. Medicare beneficiaries aged 65 years and older during the COVID-19 pandemic.

**Methods:**

This cross-sectional study analyzed Medicare beneficiaries aged 65 years and older using data from the Medicare Current Beneficiary Survey, Winter 2021 COVID-19 Supplement ($n=9,185$). We identified variables that were associated with telehealth offered by primary care physicians and beneficiaries’ access to the Internet through a multivariate classification analysis utilizing Random Forest machine learning techniques.

**Findings:**

For study participants interviewed by telephone, 81.06% of primary care providers provided telehealth services, and 84.62% of the Medicare beneficiaries had access to the Internet. The survey response rates for each outcome were 74.86% and 99.55% respectively. The two outcomes were positively correlated ($\chi ^2=268.58,\,p<.001$). The Our machine learning model predicted the outcomes accurately utilizing 44 variables. Residing area and race/ethnicity were most informative for predicting telehealth coverage, and Medicare-Medicaid dual eligibility and income were most informative for predicting Internet access. Other strong correlates included age, ability to access basic needs and certain mental and physical health conditions. Interactions were found among statuses of residing area, age, Medicare Advantage and heart conditions that intensified the disparity of outcomes.

**Conclusions:**

We found that telehealth offered by providers likely increased during the COVID-19 pandemic for older beneficiaries, providing important access to care for certain subgroups. Policymakers must continue to identify effective means of delivering telehealth services, modernize the framework of regulatory, accreditation and reimbursement, and address disparities in access to telehealth with a particular focus on underserved communities.

**Supplementary Information:**

The online version contains supplementary material available at 10.1186/s12889-023-15263-0.

## Background

The COVID-19 pandemic has been a catalyst in increasing the utilization of telehealth services. It has triggered the rapid shift from traditional in-person visits to a hybrid model of in-person and telebehavioral health visits [1, 2], especially if the physician’s office presents logistical barriers caused by the pandemic such as inconvenient clinic hours [3, 4]. The use of telehealth pre-COVID-19 was very limited in Medicare fee-for-service (FFS) and only available to beneficiaries enrolled in Medicare Advantage via national telehealth platforms. However, during the COVID-19 pandemic, Medicare FFS reimbursement for telehealth visits was the same as in-person visits. Providers also quickly set up telehealth in response to the public health emergency (PHE). These changes removed the rural-only geographic restrictions, expanded services eligible for telehealth and enabled widespread use of telehealth among Medicare FFS beneficiaries during the COVID-19 PHE [5]. Older adults, particularly those with weak immune systems, are most at risk for underlying conditions that may lead to more severe COVID-19 illness or complications [6, 7]. When older adults tend to delay or forego traditional in-person health care, telehealth services should be utilized because of their safety in providing healthcare services and mitigating the risks of infection [8, 9]. However, there are policy and payment restrictions identifying where beneficiaries could receive these services and which providers could be paid to deliver them [10, 11]. As means to support vulnerable older patients, exploring the use of telehealth is important to foster health system resilience and provide policy implications related to extending telehealth coverage under traditional Medicare.

In previous studies, pre-COVID disparities in the use of telehealth were reported to be associated with age, race/ethnicity, income, and geography [12–14]. With the emergence of COVID-19, efforts have been made to expand telehealth options to eliminate racial, residential and age disparities [15, 16]. There are studies reporting that the use of telehealth has been maximized during the initial stage of the COVID-19 pandemic [17–19]. For instance, the mean number of patients with virtual visits per month at Mayo Clinic increased from 3.5 (July 2019 to February 2020) to 172 (March to December 2020) [11, 20]. The budding literature on telehealth utilization during the pandemic emphasized that patients had an overall positive view of and were satisfied with telehealth [21–23]. However, no telehealth program can be created overnight by all primary care physicians (PCP) and specialists (SP). In previous studies on telehealth utilization, little attention has been paid to Medicare beneficiaries aged 65 years and older. Along with telemedical innovations and vaccine administration [24, 25], understanding the experiences of these Medicare beneficiaries is essential for policymakers to assess the capacity to treat patients and make diverse contributions to telehealth.

In the present study, the objective was to focus on self-reported telehealth utilization and access to the Internet among Medicare beneficiaries aged 65 years and older during the COVID-19 pandemic. Because these two outcomes could be potentially correlated, we conducted a multivariate classification analysis. Independant variables included socio-demographic factors, personal experiences with COVID-19, economic and mental effects of the pandemic, Non-COVID-19 health status and interview time. There is a high dimension of variables with complex relationships, so we decide to use machine learning approaches. Nowadays, scientists and researchers used the machine learning and deep learning models in several applications including agriculture [26, 27], environment [28–34], text sentiment analyses [35], cyber security [36–38],and medicine [39]. Since there are many correlated variables with missing values in our dataset, we utilized Random Forest machine learning techniques for the multivariate classification analysis [40, 41].

## Methods

### Data source and variables

We used the survey data from the Medicare Current Beneficiary Survey (MCBS) Winter 2021 COVID-19 Supplement, administered by telephone interview conducted by trained and certified NORC at the University of Chicago field interviewers from February through April 2021. As a continuous, multipurpose survey, the MCBS is sponsored by the Centers for Medicare & Medicaid Services (CMS) in the U.S. The original MCBS primarily focuses on outcomes such as changes in health status, spending down to Medicaid eligibility, impacts of the Medicare program, changes in satisfaction with care, and the usual source of care. With the emergence of COVID-19, CMS was uniquely positioned to use the MCBS as a vehicle to collect vital information on how the pandemic is impacting the Medicare population, and made the data publicly available at the MCBS COVID-19 Supplement Public Use File. This is a nationally representative survey of all Medicare beneficiaries, and we chose Medicare beneficiaries aged 65 years and older as our target population.

We conducted descriptive analyses as an overview of patterns of telehealth offerings and access to the Internet (see Table S1 for the items in the questionnaire) using variables including socio-demographic factors, personal experiences with COVID-19, economic and mental effects of the pandemic, and non-COVID-19 health status. We also conducted a multivariate classification analysis to detect significant predictors.

### Statistical analysis

All analyses adopted sampling weights provided by the MCBS to give nationally representative estimates. All percentages and proportions were calculated using survey weights. A weighted chi-squared test was used for the descriptive overview of each predictor. Random Forest [40] model was applied for the multivariate classification analysis, which is a modern machine learning technique that has been utilized to select replicable sets of exploratory factors from a large number of predictors [42–47]. Because this method is completely nonparametric without any restrictive underlying model assumptions, nonlinear and complex interrelationships can be robustly accounted for. After fitting the Random Forest model, variable importance (VIMP) [40, 41] and partial plots [48, 49] were adopted to identify and depict variables that are associated with the outcomes after adjusting for all the other variables. We select informative variables as those with positive VIMP estimates whose *P* values are less than 0.05. The VIMP can be interpreted as the increase in the misclassification error when the predictor of interest is randomly permutated into a noise variable. Negative VIMP values categorize “noisy” variables that degrade model accuracy. The VIMP and misclassification errors are calculated in a cross-validated fashion using the data proportion that is not used for fitting the model (a forest of classification trees is “grown” from bootstrap samples of the original dataset, leaving an average of 37% of the data not sampled, which is referred to as out-of-bag data).

We implemented weighted chi-squared tests and the Random Forest model in the open-source R software using the weights [50] and randomForestSRC [51, 52] packages respectively. The function wtd.chi.sq from the weights package was used for conducting weighted chi-squared tests. From the randomForestSRC package, the function rfsrc was used with 1000 trees and the function tune and parameters na.action = “na.impute” and case.wt were used for tuning the model, for imputing missing values of independent variables [53, 54] and for survey weighting, respectively; then the function subsample was used for estimating inferences of VIMP with default settings using 1000 subsamples [55]. There are two major tuning parameters, the number of variables to possibly split at each node (mtry) and the minimum size of terminal node (nodesize); model performance is evaluated from different combinations of mtry and nodesize to determine the final optimized forest (see Fig. S1 for details). Maximal subtree analysis was used for detecting interactions between predictors [56, 57] (see Fig. S2 for the heatmap). Partial plots were generated by setting the partial parameter in the plot.variable function [58]. The statistical significance level was set at .05.

## Results

**Fig. 1:**
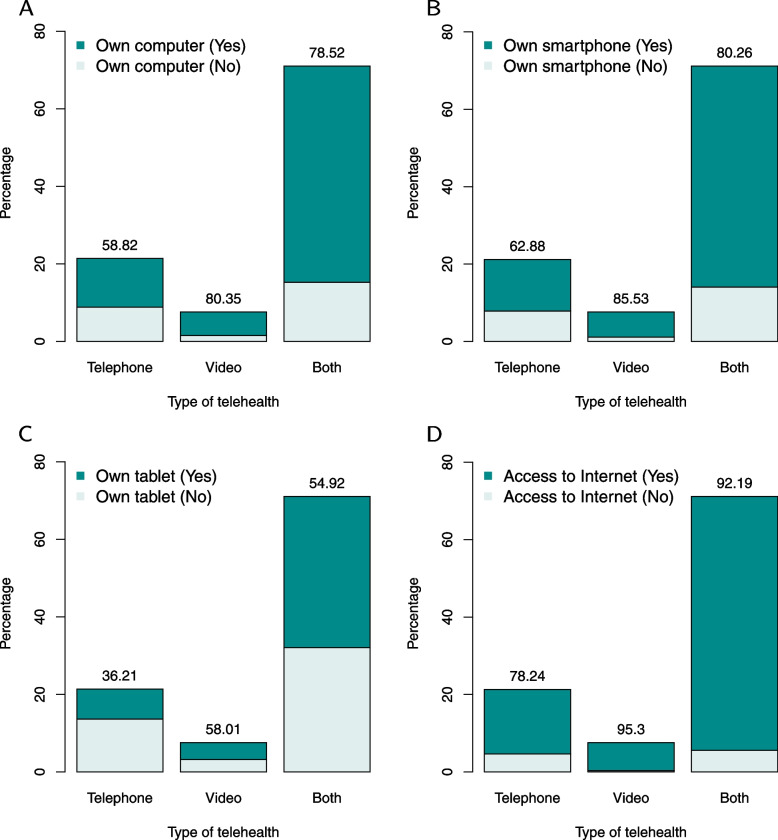
Distribution of type of telehealth offered and electronic device usage. The survey-weighted percentage of the “yes” category is listed on the top. **A** The association between type of telehealth and owning a computer. **B** The association between type of telehealth and owning a smartphone. **C** The association between type of telehealth and owning a tablet. **D** The association between type of telehealth and Internet access

The MCBS Public Use File contains 11,107 Medicare beneficiaries in total, among which 9,185 beneficiaries aged 65 years and older (82.70%, survey-weighted 85.38%) were included in this study (survey-weighted $n=$ 49.00 million). For answering whether PCP offered telehealth appointments, 1,964 and 1 beneficiaries reported “don’t know” and “refused”, respectively, with 344 inapplicable/missing data see Table S2 for more information on the missing values. For answering whether they had access to the Internet, 40 and one beneficiaries reported “don’t know” and “refused,” respectively. These categories were inappropriate for implementing the weighted chi-squared tests, therefore discarded in the descriptive analysis for both outcomes and independent variables. The survey response rates for each outcome were 74.86% and 99.55% respectively. From the yes and no categories, 81.06% and 84.62% respondents reported telehealth coverage and Internet access respectively. The two outcomes were positively correlated ($\chi ^2=268.58,\,p<.001$). The type of telehealth offered was summarized as “telephone”, “video” and “both”, whose survey-weighted percentages were 21.31, 7.56 and 71.14, respectively. The association between the type of telehealth offered and electronic device usage is shown in Fig. 1. Access to the Internet and owning electronic devices positively relate to the categories “video” and “both”.

### Descriptive analysis

The main characteristics of the sample are displayed in Table 1 with frequency, survey-weighted percentage and significant level of test statistics. There are 43 variables, including ten socio-demographic variables, two variables describing personal experiences with COVID-19, seven variables describing the economic and mental effects of the pandemic, and 24 variables recording non-COVID-19 health status.

Among socio-demographic factors, 7 of 10 were significantly associated with both outcomes, including age, race/ethnicity, residing area (metro vs non-metro), census region, income, use of a language other than English at home (shown as non-English in Table 1) and Medicare-Medicaid dual eligibility. The male group and the group without prescription drug coverage (Part D plan) significantly tended to have access to the Internet. The status of Medicare Advantage (MA) also played a significant role. The two variables describing personal experiences with COVID-19, which recorded COVID-19 test and COVID-19 antibody test results, were not significantly associated with the outcomes. Among factors describing the economic and mental effects of the pandemic, 3 of 7 were significantly associated with both outcomes. Beneficiaries who felt more financially secure, more stressed and less socially connected were more likely to have access to telehealth and the Internet; beneficiaries with access to the Internet were those who were able to pay rent/mortgage as well as get food and home supplies. Most of the 24 variables recording non-COVID-19 health status were significantly associated with either of the outcomes.

**Table 1:** Descriptive analysis of telehealth coverage and Internet access

		Number (Survey-weighted percentage^†^)					
			Coverage of telehealth		Access to the Internet		
Variable	Category	Frequency	Yes	No	Yes	No	Sig^‡^
Overall	Overall	9,185	5398 (81)	1478 (19)	7255 (85)	1889 (15)	
Age	65 - 74	3919 (60)	2504 (84)	496 (16)	3452 (90)	457 (10)	***+++
	74+	5266 (40)	2894 (76)	982 (24)	3803 (76)	1432 (24)	
Gender	Male	4042 (45)	2374 (81)	672 (19)	3304 (86)	725 (14)	+++
	Female	5143 (55)	3024 (81)	806 (19)	3951 (83)	1164 (17)	
Race/ethnicity	White non-Hispanic	7085 (78)	4195 (83)	1012 (17)	5958 (89)	1103 (11)	***+++
	Black non-Hispanic	723 (8)	389 (72)	175 (28)	428 (67)	286 (33)	
	Hispanic	932 (8)	559 (74)	197 (26)	526 (65)	400 (35)	
	Other/Unknown	445 (6)	255 (76)	94 (24)	343 (83)	100 (17)	
Metro residence	Metro	7163 (81)	4456 (83)	1041 (17)	5777 (86)	1354 (14)	***+++
	Non-metro	2020 (19)	940 (71)	437 (29)	1477 (78)	534 (22)	
Region	Northeast	1650 (18)	1010 (82)	244 (18)	1297 (85)	351 (15)	***+++
	Midwest	2017 (22)	1158 (84)	268 (16)	1627 (85)	383 (15)	
	South	3519 (39)	1914 (76)	712 (24)	2648 (81)	856 (19)	
	West	1998 (22)	1315 (86)	254 (14)	1683 (90)	298 (10)	
Income	Less than $25,000	2577 (25)	1315 (72)	579 (28)	1421 (63)	1133 (37)	***+++
	$25,000 or more	6251 (75)	3917 (84)	821 (16)	5608 (92)	628 (8)	
Non-English	Yes	1123 (11)	657 (75)	232 (25)	679 (71)	439 (29)	***+++
	No	8055 (89)	4737 (82)	1243 (18)	6571 (86)	1448 (14)	
Medicare-Medicaid dual eligibility	Full	692 (6)	376 (70)	166 (30)	290 (49)	396 (51)	***+++
	Nondual	8062 (90)	4809 (82)	1200 (18)	6729 (88)	1304 (12)	
	Partial	237 (2)	114 (72)	57 (28)	129 (61)	104 (39)	
	QMB only	194 (2)	99 (68)	55 (32)	107 (60)	85 (40)	
Medicare Advantage (MA)	No MA enrollment	5033 (58)	2921 (81)	801 (19)	4135 (87)	875 (13)	+++
	Partial-year MA	165 (3)	94 (86)	20 (14)	123 (82)	42 (18)	
	Full-year MA	3983 (38)	2380 (81)	657 (19)	2993 (81)	972 (19)	
Part D plan	Yes	7345 (77)	4294 (81)	1211 (19)	5701 (83)	1608 (17)	+++
	No	1836 (23)	1101 (82)	267 (18)	1550 (89)	281 (11)	
Positive COVID-19 test	Yes	340 (12)	229 (86)	44 (14)	267 (85)	71 (15)	
	No	2579 (88)	1617 (82)	399 (18)	2120 (87)	450 (13)	
	No results yet	19 (1)	14 (82)	3 (18)	17 (96)	2 (4)	
Positive COVID19 antibody test	Yes	58 (20)	39 (87)	9 (13)	51 (94)	7 (6)	
	No	223 (78)	156 (88)	26 (12)	200 (93)	23 (7)	
	No results yet	7 (2)	4 (100)	0 (0)	7 (100)	0 (0)	
Able to pay rent or mortgage	Able	5237 (60)	3153 (82)	836 (18)	4145 (85)	1067 (15)	++
	Unable	96 (1)	58 (76)	20 (24)	63 (73)	33 (27)	
	Not needed	3821 (39)	2174 (80)	615 (20)	3026 (85)	780 (15)	
Able to get food	Able	8902 (97)	5248 (81)	1422 (19)	7063 (85)	1801 (15)	+++
	Unable	150 (2)	82 (73)	33 (27)	112 (83)	35 (17)	
	Not needed	117 (1)	62 (79)	18 (21)	70 (70)	47 (30)	
Able to get home supplies	Able	8788 (96)	5170 (81)	1409 (19)	6968 (85)	1780 (15)	+++
	Unable	212 (2)	135 (82)	35 (18)	170 (85)	42 (15)	
	Not needed	168 (2)	87 (76)	29 (24)	107 (74)	60 (26)	
Feel financially secure	More secure	442 (6)	291 (86)	53 (14)	388 (91)	50 (9)	*+++
	Less secure	836 (11)	484 (79)	150 (21)	638 (81)	193 (19)	
	About the same	6936 (84)	4072 (82)	1074 (18)	5677 (87)	1230 (13)	
Feel stressed	More stressed	2809 (37)	1800 (84)	378 (16)	2380 (89)	414 (11)	***+++
	Less stressed	353 (5)	212 (81)	64 (19)	285 (87)	66 (13)	
	About the same	5037 (59)	2835 (80)	833 (20)	4036 (85)	981 (15)	
Feel lonely or sad	More lonely or sad	1677 (21)	1021 (82)	255 (18)	1406 (87)	259 (13)	
	Less lonely or sad	283 (4)	172 (81)	50 (19)	234 (87)	49 (13)	
	About the same	6226 (76)	3639 (82)	968 (18)	5044 (86)	1156 (14)	
Feel socially connected	More connected	778 (10)	476 (82)	126 (18)	648 (87)	127 (13)	***+++
	Less connected	3193 (40)	2006 (84)	431 (16)	2789 (91)	391 (9)	
	About the same	4239 (50)	2367 (80)	716 (20)	3275 (83)	942 (17)	
Weak immune system (treatment/drug)	Yes	394 (4)	269 (83)	58 (17)	312 (84)	82 (16)	
	No	8656 (96)	5060 (81)	1396 (19)	6854 (85)	1761 (15)	
Weak immune system (health condition)	Yes	1190 (14)	804 (84)	154 (16)	947 (84)	241 (16)	**
	No	7742 (86)	4444 (80)	1297 (20)	6116 (85)	1590 (15)	
Weak immune system (any reason)	Yes	1301 (15)	873 (85)	170 (15)	1036 (85)	263 (15)	**
	No	7847 (85)	4508 (80)	1304 (20)	6197 (85)	1611 (15)	
Hypertension/high BP	Yes	6163 (64)	3659 (80)	1065 (20)	4725 (82)	1409 (18)	***+++
	No	3013 (36)	1736 (83)	410 (17)	2522 (89)	479 (11)	
Myocardial infarction	Yes	982 (10)	578 (76)	186 (24)	728 (80)	250 (20)	***+++
	No	8185 (90)	4809 (82)	1290 (18)	6512 (85)	1636 (15)	
Angina pectoris/CHD	Yes	875 (9)	534 (81)	142 (19)	715 (86)	157 (14)	
	No	8252 (91)	4836 (81)	1323 (19)	6499 (85)	1715 (15)	
Congestive heart failure	Yes	594 (6)	338 (74)	128 (26)	436 (79)	156 (21)	***+++
	No	8563 (94)	5047 (82)	1343 (18)	6804 (85)	1720 (15)	
Other heart conditions, eg valve/rhythm	Yes	2264 (22)	1366 (81)	372 (19)	1807 (85)	447 (15)	
	No	6902 (78)	4021 (81)	1103 (19)	5433 (85)	1438 (15)	
Stroke or brain hemorrhage	Yes	909 (9)	546 (79)	161 (21)	639 (76)	262 (24)	+++
	No	8271 (91)	4849 (81)	1317 (19)	6612 (85)	1626 (15)	
High cholesterol	Yes	6117 (65)	3689 (81)	1000 (19)	4827 (84)	1262 (16)	
	No	3033 (35)	1694 (81)	472 (19)	2404 (85)	616 (15)	
Cancer (non-skin)	Yes	1940 (20)	1202 (82)	304 (18)	1541 (84)	393 (16)	
	No	7235 (80)	4191 (81)	1172 (19)	5706 (85)	1494 (15)	
Alzheimers/dementia	Yes	395 (3)	230 (79)	75 (21)	222 (61)	171 (39)	+++
	No	8788 (97)	5168 (81)	1403 (19)	7031 (85)	1718 (15)	
Depression	Yes	1919 (21)	1211 (82)	309 (18)	1472 (83)	438 (17)	
	No	7246 (79)	4177 (81)	1165 (19)	5769 (85)	1445 (15)	
Osteoporosis or soft bones	Yes	1879 (19)	1154 (81)	294 (19)	1466 (84)	404 (16)	
	No	7262 (81)	4223 (81)	1172 (19)	5764 (85)	1466 (15)	
Broken hip	Yes	355 (3)	205 (82)	58 (18)	238 (74)	116 (26)	+++
	No	8823 (97)	5190 (81)	1419 (19)	7014 (85)	1769 (15)	
Emphysema/asthma/COPD	Yes	1657 (17)	1036 (81)	276 (19)	1284 (82)	364 (18)	++
	No	7516 (83)	4357 (81)	1200 (19)	5964 (85)	1520 (15)	
Diabetes/high blood sugar	Yes	2808 (30)	1789 (83)	441 (17)	2143 (82)	651 (18)	*+++
	No	6359 (70)	3603 (80)	1031 (20)	5098 (86)	1235 (14)	
Any arthritis	Yes	2790 (64)	1681 (79)	494 (21)	2103 (81)	671 (19)	+
	No	1464 (36)	856 (82)	238 (18)	1157 (84)	299 (16)	
Any heart condition	Yes	3253 (32)	1945 (80)	550 (20)	2548 (84)	691 (16)	
	No	5901 (68)	3436 (82)	924 (18)	4685 (85)	1189 (15)	
Any osteoporosis or broken hip	Yes	2085 (20)	1272 (81)	323 (19)	1602 (83)	473 (17)	++
	No	7056 (80)	4106 (81)	1143 (19)	5630 (85)	1395 (15)	
Ever smoke cigarette/cigar/pipe	Yes	5148 (56)	3024 (81)	843 (19)	4183 (86)	942 (14)	+++
	No	4034 (44)	2371 (81)	635 (19)	3070 (83)	946 (17)	
Currently smoke cigarette/cigar/pipe	Yes	688 (16)	381 (78)	132 (22)	522 (80)	162 (20)	*+++
	No	4456 (84)	2641 (81)	710 (19)	3657 (87)	780 (13)	
Ever used e-cigarette	Yes	484 (6)	284 (80)	78 (20)	414 (88)	67 (12)	+
	No	8687 (94)	5109 (81)	1397 (19)	6835 (84)	1814 (16)	
Smoke e-cigarette now	Yes	63 (15)	38 (75)	12 (25)	57 (92)	6 (8)	
	No	421 (85)	246 (81)	66 (19)	357 (87)	61 (13)	

^†^Categories of “inapplicable/missing”, “don’t know”, “not ascertained”, and “refused” were excluded in calculating percentages and weighted chi-squared statistics

^‡^Sig indicates significant level according to *P* values: when the outcome is telehealth coverage, * for $p\le$ 0.05, ** for $p\le$ 0.01, *** for $p\le$ 0.001; when the outcome is Internet access, + for $p\le$ 0.05, ++ for $p\le$ 0.01, and +++ for $p\le$ 0.001

### Multivariate classification analysis

**Fig. 2:**
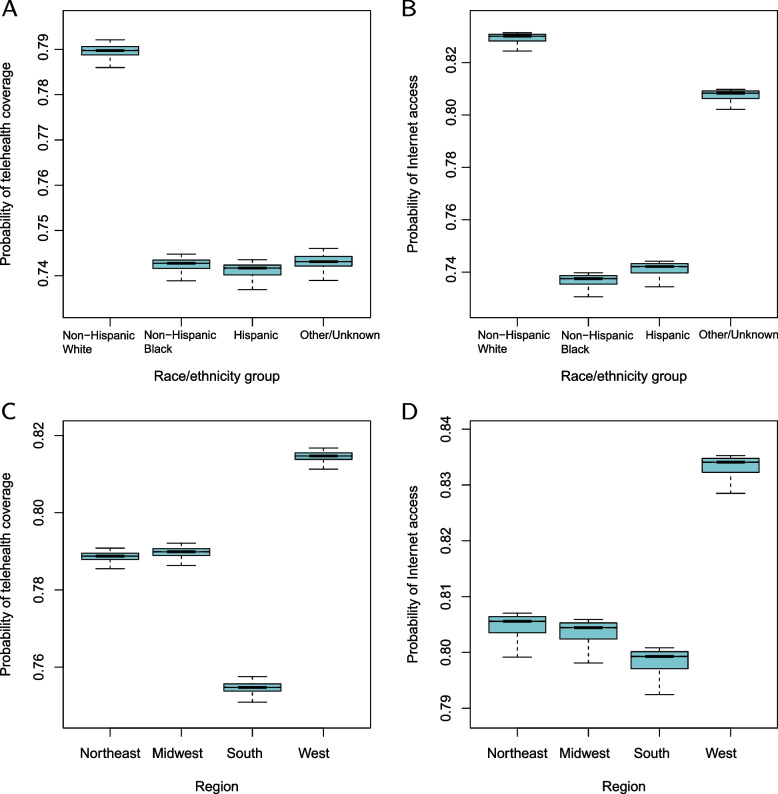
Random Forest estimated probabilities of outcomes plotted against candidate variables. **A** The association between race/ethnicity and telehealth coverage. **B** The association between race/ethnicity and Internet access. **C** The association between region and telehealth coverage. **D** The association between region and Internet access

Important variables for predicting both outcomes were identified by machine learning using the Random Forest multivariate classification model, and the results are shown in Table 2 and Fig. S2. Only yes and no responses of the outcomes were included in the Random Forest model ($n=6848,p=44$). All variables in Table 1 were added to the classification model with a variable recording interview date added. The Random Forest classification model predicted the outcomes accurately: the out-of-bag misclassification error is 21.22% for predicting telehealth coverage and 17.60% for Internet acess. The complete list of VIMP for Table 2 can be found in Appendix Table S3. We also used Internet access as an additional predictor for the outcome telehealth provided by PCP and Table S4 showed significant predictors, among which Internet access is the strongest predictor. Table 1 consists of stacked contingency tables of variables, and the first two rows of each contingency table for each variable were used for calculating an odds ratio (OR) with survey weights to demonstrate the direction of effects. Table 2 presents the estimate, standard error (SE), and the *P* value of VIMP, followed by the survey-weighted OR. A large estimate of VIMP indicates a variable that is strongly associated with the corresponding outcome, while a negative estimate indicates a noise variable. An OR greater than 1 indicates a positive association between the first category of the variable and the corresponding outcome, compared with its second category, while an OR less than 1 indicates a negative association. For variables with yes and no responses, an OR greater than 1 indicates a positive association since the first category is always for the yes response. The effects of three informative multifactorial categorical variables are shown in Fig. 2 for race/ethnicity and region and Fig. S3 for Medicare Advantage.

#### Coverage of telehealth

We detected 14 variables significantly associated with telehealth coverage after adjusting for other variables. Residing area (metro residence, VIMP = 2.00, SE = 0.24, $p<$ .001, OR = 2.00) and race/ethnicity (VIMP = 1.14, SE = 0.27, $p<$ .001, OR = 1.92) were the most informative factors (see Table 2), indicating that non-hispanic white and metro residence were more likely to have coverage of telehealth. In addition, the relatively young group (VIMP = 0.10, SE = 0.02, $p<$ .001, OR = 1.65) and high income group (VIMP = 0.10, SE = 0.05, $p=$ .026, OR = 0.49) tended to have higher coverage. Beneficiaries who had positive COVID-19 test (VIMP = 0.25, SE = 0.10, $p=$ .005, OR = 1.31) and were able to get food (VIMP = 0.99, SE = 0.30, $p=$ .001, OR = 1.58) and pay rent/mortgage (VIMP = 0.47, SE = 0.21, $p=$ .011, OR = 1.39) were more likely to have telehealth coverage.

In terms of mental effects of the pandemic and non-COVID-19 health status or habit, beneficiaries with telehealth coverage tended to feel lonely or sad (VIMP = 0.38, SE = 0.13, $p=$ .002, OR = 1.05) and have depression (VIMP = 0.05, SE = 0.01, $p<$ .001, OR = 1.08). Beneficiaries who had coverage of telehealth were more likely with high cholesterol (VIMP = 0.12, SE = 0.01, $p<$ .001, OR = 1.04), but no heart conditions such as myocardial infarction, angina pectoris/coronary heart disease (CHD), congestive heart failure (see Table S3) or abnormal valve/rhythm (VIMP = 0.01, SE = 0.01, $p=$ .026, OR = 0.96); they were with low probability of having any arthritis (VIMP = 0.06, SE = 0.01, $p<$ .001, OR = 0.83) or reporting e-cigarette usage (ever used, VIMP = 0.15, SE = 0.07, $p=$ .016, OR = 0.92; smoke now, VIMP = 0.61, SE = 0.09, $p<$ .001, OR = 0.72)

#### Access to the internet

Among the 18 variables listed in Table 1, 11 variables were significantly associated with access to the Internet. The two most informative factors are Medicare-Medicaid dual eligibility (VIMP = 9.78, SE = 1.16, $p<$ .001, OR = 0.13) and income (VIMP = 3.83, SE = 0.49 $p<$ .001, OR = 0.14), indicating that nondual-eligible beneficiaries (not eligible for Medicaid benefits) and beneficiaries with higher income were more likely to have access to the Internet. In addition, the non-hispanic white group (VIMP = 2.05, SE = 0.44, $p<$ .001, OR = 3.85) and relatively young group (VIMP = 0.96, SE = 0.10, $p<$ .001, OR = 2.74) tended to have access to the Internet. Beneficiaries with Internet access were those who were able to get food (VIMP = 0.83, SE = 0.24, $p<$ .001, OR = 1.18) but more likely to feel lonely or sad (VIMP = 0.14, SE = 0.07, $p=$ .018, OR = 1.06) .

In terms of non-COVID-19 health status or habit, beneficiaries with Internet access tended to report e-cigarette usage (ever used, VIMP = 0.15, SE = 0.06, $p=$ .008, OR = 1.32) but do not have weak immune system due to health conditions (VIMP = 0.01, SE = 0.01, $p=$ .039, OR = 0.98). Although beneficiaries reporting any heart condition had lower probability of Internet access, those with angina pectoris/CHD (VIMP = 0.13, SE = 0.02, $p<$ .001, OR = 1.16) and with other heart conidtion such as abnormal valve/rhythm (VIMP = 0.04, SE = 0.01, $p<$.001, OR = 1.02) were more likely to have access to the Internet.

**Table 2:** Informative variables for predicting telehealth coverage and Internet access from Random Forest analyses

	Coverage of telehealth				Access to the Internet				
Variable	Est	SE	*P* value	OR^§^	Est	SE	*P* value	OR^§^	Sig^‡^
Age	0.10	0.02	0.000	1.65	0.96	0.10	0.000	2.74	***+++
Race/ethnicity	1.14	0.27	0.000	1.92	2.05	0.44	0.000	3.85	***+++
Metro residence	2.00	0.24	0.000	2.00	-0.04	0.03	0.878	1.70	***
Income	0.10	0.05	0.026	0.49	3.83	0.49	0.000	0.14	*+++
Medicare-Medicaid dual eligibility	0.50	0.53	0.172	0.50	9.78	1.16	0.000	0.13	+++
Positive COVID-19 test	0.25	0.10	0.005	1.31	0.03	0.14	0.411	0.82	**
Able to pay rent/mortgage	0.47	0.21	0.011	1.39	0.24	0.26	0.172	2.13	*
Able to get food	0.99	0.30	0.001	1.58	0.83	0.24	0.000	1.18	**+++
Feel lonely or sad	0.38	0.13	0.002	1.05	0.14	0.07	0.018	1.06	**+
Weak immune system due to health cond	-0.06	0.01	1.000	1.34	0.01	0.01	0.039	0.98	+
Angina pectoris/CHD	-0.01	0.02	0.724	0.98	0.13	0.02	0.000	1.16	+++
Other heart conditions, eg valve/rhythm	0.01	0.01	0.026	0.96	0.04	0.01	0.000	1.02	*+++
High cholesterol	0.12	0.01	0.000	1.04	-0.02	0.01	0.999	0.95	***
Depression	0.05	0.01	0.000	1.08	-0.05	0.01	1.000	0.88	***
Any arthritis	0.06	0.01	0.000	0.83	-0.04	0.01	1.000	0.82	***
Any heart condition	0.01	0.01	0.141	0.89	0.05	0.01	0.000	0.90	+++
Ever used e-cigarette	0.15	0.07	0.016	0.92	0.15	0.06	0.008	1.32	*++
Smoke e-cigarette now	0.61	0.09	0.000	0.72	0.04	0.03	0.152	1.60	***

Est and SE indicate estimation and standard error for Random Forest variable importance (VIMP)

^§^OR indicates the survey-weighted odds ratio indicating the direction of effects: if the value is larger than one, the first category of the variable in Table 1 is more likely with a positive outcome than the second category. For example, the odds ratio of age is 1.65, indicating that the 65 to 74 age group was more likely with telehealth coverage than the over-74 age group.

^‡^Sig indicates significant level according to *P* values of VIMP: when the outcome is telehealth coverage, * for $p\le$ 0.05, ** for $p\le$ 0.01, *** for $p\le$ 0.001; when the outcome is Internet access, + for $p\le$ 0.05, ++ for $p\le$ 0.01, and +++ for $p\le$ 0.001

#### Variable interactions

**Fig. 3:**
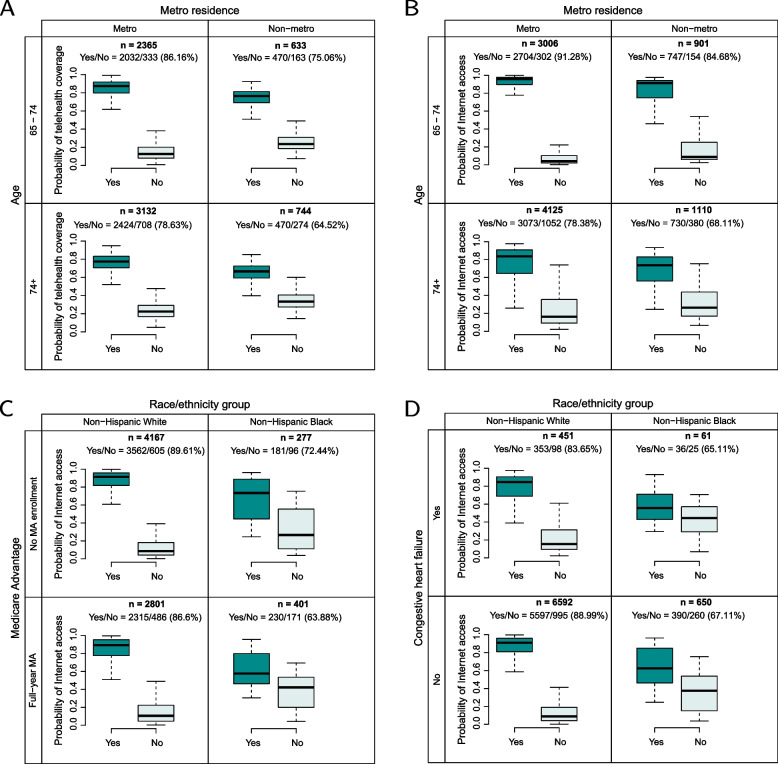
Interactions of variables for predicting the probabilities of telehealth coverage and Internet access. The survey-weighted proportions of positive outcomes are listed in the parentheses. **A** The interaction between residing area and age for predicting telehealth coverage. **B** The interaction between residing area and age for predicting Internet access. **C** The interaction between race/ethnicity and Medicare Advantage (MA) for predicting Internet access. **D** The interaction between race/ethnicity and congestive heart failure (ever) for predicting Internet access

We found three pairs of variables that intensified the disparity in both outcomes among combined categories. The interaction between residing area and age is demonstrated in Fig. 3A and B for the two outcomes. The 65 to 74 age group with the status of metro residence had higher probabilities of telehealth coverage and Internet access (86.16% and 91.28%) than the over-74 age group with the status of non-metro residence (64.52% and 68.11%). The interaction between race/ethnicity and Medicare Advantage is demonstrated in Fig. 3C for Internet access and Fig. S3A for telehealth coverage. The non-Hispanic white group with no Medicare Advantage enrollment had higher probabilities of Internet access (89.61%) and telehealth coverage (83.43%) than the non-Hispanic black group (72.44% and 67.28%). The non-Hispanic white group without congestive heart failure (ever) also had higher probabilities of Internet access (88.99% shown in Fig. 3D) and telehealth coverage (83.58% shown in Fig. S4B) than the non-Hispanic black group with congestive heart failure (65.11% and 67.45%).

## Discussion

This study set out to investigate the use of telehealth among older adults during the pandemic in the U.S. Specifically, we aimed to model telehealth coverage offered by PCP and Internet access reported by Medicare beneficiaries aged 65 Years and older during the Winter of 2021. Utilizing nationally representative survey data, we examined patterns in coverage of telehealth and access to the Internet during the COVID-19 pandemic. First, we identified about four-fifth of older beneficiaries who reported telehealth coverage and Internet access during the pandemic. Our estimate for telehealth coverage was about the same as the estimate from prior work on overall beneficiaries (80.2%) [11]. Compared with Internet access, the low survey response rate for telehealth coverage indicates that older people may be accustomed to seeing a doctor in person and less comfortable with technology [59], possibly due to physical limitations, such as impaired vision or manual dexterity [60]. Our work adds to the existing literature by identifying factors contributing to telehealth usage in a nationally representative population with stable health insurance during the COVID-19 pandemic. Strong correlates, such as Medicare beneficiaries’ age, race/ethnicity, income, Medicare-Medicaid dual eligibility, ability to access basic needs, and certain mental and physical health conditions, are consistent with some prior studies [61–66].

We found that self-reported telehealth coverage is strongly associated with the result of beneficiaries’ active COVID-19 test, indicating increased awareness of telehealth among COVID-19 patients and the importance of enhancing telehealth coverage for containing the pandemic. We found that Internet access was positively related to telehealth coverage and associated with the type of telehealth, indicating that investment in technology infrastructure could have a significant impact on improving access to healthcare. Although our primary outcome, telehealth provided by PCP, would be affected more by provider factors than patient factors, the survey does not offer much information on provider factors. Therefore, we could only use patient factors in this paper. Some patient characteristics could be directly associated with whether a provider offers a visit to be delivered via telehealth, such as whether the patient is suspected of having COVID-19, or is considered a high-risk patient due to underlying health conditions. Some patient characteristics, such as MA enrollment or race/ethnicity, could be indirectly related to the outcome since they are confounded with some unobserved provider factors, especially under the condition that no telehealth program can be created overnight. For example, before the COVID-19 pandemic, if telehealth was only available to beneficiaries enrolled in MA, then during the pandemic, even MA itself is no longer associated with telehealth access anymore, both beneficiaries enrolled in MA and their providers tend to have more awareness of telehealth with a telehealth program that was already created.

We attempted to identify potential barriers to the implementation of telehealth services during the COVID-19 pandemic. Our findings confirmed and expanded previous results that identified barriers to access and utilization of telemedicine, such as the age of the patient, racial/ethnic disparities, and type of community and geographic location [67, 68]. Our results suggested that beneficiary who is less likely to have access to telehealth from PCP tends to be someone who is a non-metro residence, with a lower income, or without Internet access. Telehealth utilization in rural areas, particularly the Southern regions of the country, has previously been limited [69, 70]. However, the regional differences in older beneficiaries’ access to the Internet were not as large. Although our descriptive statistics show that males were more likely to have Internet access, this difference is not significant in our classification model after adjusting for other variables. Previous studies found that females utilized telehealth services more than males [71], while we observed similar awareness of primary care virtual appointments between females and males in U.S. Medicare beneficiaries aged 65 years and older.

Before the COVID-19 pandemic, telehealth reimbursement was limited to the management of chronic conditions [11]. Limited reimbursement may constrain the widespread use of telehealth. We found that several mental and physical health conditions were significantly associated with telehealth coverage, indicating that such limitations may still exist for certain chronic conditions in the wake of the pandemic. Although several health conditions were negatively associated with telehealth coverage, we found that depression was positively associated with telehealth coverage, which may reflect the parity between mental health coverage and coverage for other medical conditions.

Compared with the existing literature, we analyzed related factors in a more inclusive fashion to identify main effects and complex interactions. We believe that after adjustment for different factors, the discoveries of the most informative ones could be more consistent and reproducible. On the other hand, adding more factors brings risks of multicollinearity and missing data, which causes the problem of convergence for parametric statistical models. We tried classical logistic regression and lasso penalized logistic regression for this dataset, which did not converge due to a large amount of missing values on the predictors. When the goal is to rank the factors which are highly correlated and interacted, nonparametric variable important indices [41, 72, 73], instead of odds ratios or regression coefficients, may be more suitable for providing insights. The success of this predictive model was also largely attributed to the high quality of the data. Although there are many missing values, the misclassification errors are about only 20%, indicating high accuracy of missing data imputation by Random Forest [53] and low common biases related to surveys, such as recall bias. Random Forest methods have a high potential for analyzing survey data whose variables are multifactorial and interacted.

## Limitations

This study has several limitations. First, it relies upon self-reported data from a national survey limited to a relatively short period. As such, our findings may be subject to self-reporting errors and are not generalizable to older adults who are non-Medicare beneficiaries or live in long-term care facilities. We do not have data to infer if similar issues occurred in the broader population and patients on commercial insurance or uninsured. Second, our study has two types of missing data problems: the missing values for the outcome and the predictors. Although low misclassification errors indicate high accuracy of missing data imputation for the predictors by Random Forest, we have to omit the missing values for the outcome, which could bring bias to the model’s findings. Additionally, the cross-sectional nature of the data and analyses prevents assumptions of causality. As a cross-sectional study, changes in telehealth utilization were not directly assessed, but inferred based on studies of telehealth use prior to the pandemic. Furthermore, several important factors were not included in the survey, such as education attainment, information on state residency, primary diagnosis from the telehealth visit, etc. We could not evaluate effects of specific pandemic responses such as masking policies and restrictions on elective surgeries, or effects of specific regulatory issues on telemedicine such as payment, licensure, credentialing, online prescribing, medical malpractice, privacy and security, etc. Further, the mental effects of the pandemic were not evaluated via clinical mood or disorders to capture different dimensions of mental health problems.

## Conclusions

In summary, this cross-sectional survey study suggests that several barriers to telehealth utilization exist among older Medicare beneficiaries. The COVID-19 pandemic may exacerbate existing barriers for this high at-risk population. As in-person visits were being discouraged or were unavailable due to the risks of the COVID-19 virus, the need for strategies for improving telehealth utilization grows. Policymakers must continue to identify effective means of ensuring equal access and utilization of telehealth.

## Supplementary Information

**Additional file 1.**

## Data Availability

The dataset analyzed for this study can be found at https://github.com/luminwin/MCBS-PUF2021. More details about this dataset can be found in the Medicare Current Beneficiary Survey COVID-19 Supplement, available at https://www.cms.gov/research-statistics-data-systems/cms-covid-19-data-products. The questionnaire can be found at https://www.cms.gov/research-statistics-data-and-systemsresearchmcbsquestionnaires/2021-winter-supplemental-covid-19-questionnaires.
