# Supplementary material for: Telehealth utilization in U.S. Medicare beneficiaries aged 65 years and older during the COVID-19 pandemic
Source: BMC Public Health. 2023 Feb 20;23:368. doi: 10.1186/s12889-023-15263-0 (PMC9942377; doi:10.1186/s12889-023-15263-0)
Supplement: Supplementary file 1 — Additional file 1. [file 12889_2023_15263_MOESM1_ESM.pdf]

Supplementary Materials for

Telehealth Utilization in U.S. Medicare Beneficiaries Aged 65 Years and Older During the COVID-19 Pandemic

Min Lu[1]* and Xinyi Liao[1]

1 Department of Public Health Sciences, Miller School of Medicine, University of Miami, Miami, FL, USA

* Current Address: Don Soffer Clinical Research Building, Room 1057, 1120 NW 14th Street, Miami, FL 33136, USA
* Corresponding author
E-mail: m.lu6@umiami.edu (ML)

1 Tables

Table S1. Description of outcome variables.

Variable	Question*
Primary care physician (PCP) offers telehealth	Does [your/(SP)'s] usual provider offer telephone or video appointments, so that [you don't/he/she doesn't] need to physically visit their office or facility?<br><br>[IF NEEDED: Did [your/(SP)'s] provider offer to talk to [you/him/her] about [your/his/her] symptoms over the phone or video so that [you/he/she] wouldn't have to visit their office or facility?]<br><br>[IF NEEDED: Telephone appointments may include "audio-only" appointments.]
Access to the Internet	[Do you/ Does (SP)] have access to the internet?

*The response was categorized as "yes", "no", "don't know", or "refused".

Table S2. Descriptive analysis of missing values in telehealth provided by PCP.

		Number (Survey weighted percentage)				
Variable	Levels	Frequency	Yes	No	Don't know or refused or missing	Sig†
Overall	Overall	9185	5398 (61)	1478 (14)	2309 (25)	
Age	65 - 74	3919 (60)	2504 (64)	496 (12)	919 (24)	*
	74+	5266 (40)	2894 (56)	982 (18)	1390 (26)	
Gender	Male	4042 (45)	2374 (61)	672 (15)	996 (24)	
	Female	5143 (55)	3024 (60)	806 (14)	1313 (26)	
Race/ethnicity group	White non-Hispanic	7085 (78)	4195 (62)	1012 (12)	1878 (26)	***
	Black non-Hispanic	723 (8)	389 (56)	175 (22)	159 (22)	
	Hispanic	932 (8)	559 (61)	197 (21)	176 (18)	
	Other/Unknown	445 (6)	255 (58)	94 (18)	96 (24)	
Metro residence	Metro	7163 (81)	4456 (64)	1041 (13)	1666 (23)	***
	Non-metro	2020 (19)	940 (49)	437 (20)	643 (31)	
Region	Northeast	1650 (18)	1010 (63)	244 (13)	396 (24)	***
	Midwest	2017 (22)	1158 (60)	268 (12)	591 (28)	
	South	3519 (39)	1914 (57)	712 (18)	893 (25)	
	West	1998 (22)	1315 (66)	254 (11)	429 (23)	
Income	$25,000	2577 (25)	1315 (53)	579 (20)	683 (27)	**
	â‰¥ $25,000	6251 (75)	3917 (64)	821 (12)	1513 (24)	
Non-English	Yes	1123 (11)	657 (60)	232 (20)	234 (20)	***
	No	8055 (89)	4737 (61)	1243 (13)	2075 (26)	
Medicare-Medicaid dual eligibility	Full	692 (6)	376 (54)	166 (23)	150 (23)	
	Nondual	8062 (90)	4809 (62)	1200 (13)	2053 (25)	
	Partial	237 (2)	114 (53)	57 (20)	66 (27)	
	QMB only	194 (2)	99 (54)	55 (25)	40 (21)	
Medicare advantage flag	No MA enrollment	5033 (58)	2921 (60)	801 (14)	1311 (26)	***
	Partial-year MA	165 (3)	94 (56)	20 (9)	51 (35)	

	Full-year MA	3983 (38)	2380 (62)	657 (15)	946 (23)	
Part D plan flag	Yes	7345 (77)	4294 (61)	1211 (14)	1840 (25)	
	No	1836 (23)	1101 (62)	267 (14)	468 (25)	
Positive CV19 test	Yes	340 (12)	229 (71)	44 (12)	67 (17)	
	No	2579 (88)	1617 (64)	399 (14)	563 (22)	
	No results yet	19 (1)	14 (77)	3 (16)	2 (7)	
Positive CV19 antibody test	Yes	58 (20)	39 (74)	9 (11)	10 (15)	
	No	223 (78)	156 (72)	26 (10)	41 (18)	
	No results yet	7 (2)	4 (66)	0 (0)	3 (34)	
Able to pay rent/mortgage	Able	5237 (60)	3153 (62)	836 (14)	1248 (24)	***
	Unable	96 (1)	58 (66)	20 (20)	18 (14)	
	Not needed	3821 (39)	2174 (58)	615 (14)	1032 (27)	
Able to get food	Able	8902 (97)	5248 (61)	1422 (14)	2232 (25)	
	Unable	150 (2)	82 (57)	33 (21)	35 (22)	
	Not needed	117 (1)	62 (53)	18 (14)	37 (34)	
Able to get home supplies	Able	8788 (96)	5170 (61)	1409 (14)	2209 (25)	*
	Unable	212 (2)	135 (67)	35 (15)	42 (19)	
	Not needed	168 (2)	87 (52)	29 (17)	52 (31)	
Feel financially secure	More secure	442 (6)	291 (68)	53 (11)	98 (21)	
	Less secure	836 (11)	484 (59)	150 (16)	202 (25)	
	About the same	6936 (84)	4072 (61)	1074 (14)	1790 (26)	
Feel stressed	More stressed	2809 (37)	1800 (65)	378 (12)	631 (22)	***
	Less stressed	353 (5)	212 (64)	64 (15)	77 (20)	
	About the same	5037 (59)	2835 (58)	833 (15)	1369 (27)	
Feel lonely or sad	More lonely or sad	1677 (21)	1021 (62)	255 (14)	401 (24)	
	Less lonely or sad	283 (4)	172 (64)	50 (15)	61 (20)	
	About the same	6226 (76)	3639 (61)	968 (14)	1619 (26)	
Feel socially connected	More connected	778 (10)	476 (64)	126 (14)	176 (22)	***
	Less connected	3193 (40)	2006 (64)	431 (12)	756 (23)	
	About the same	4239 (50)	2367 (58)	716 (15)	1156 (27)	
Weak immune system due to treatment/drug	Yes	394 (4)	269 (72)	58 (15)	67 (13)	***
	No	8656 (96)	5060 (60)	1396 (14)	2200 (25)	
Weak immune system due to health condition	Yes	1190 (14)	804 (69)	154 (13)	232 (18)	***
	No	7742 (86)	4444 (59)	1297 (15)	2001 (26)	
Weak immune system (any reason)	Yes	1301 (15)	873 (70)	170 (13)	258 (18)	***
	No	7847 (85)	4508 (59)	1304 (14)	2035 (26)	
Hypertension/high BP	Yes	6163 (64)	3659 (62)	1065 (15)	1439 (23)	***
	No	3013 (36)	1736 (59)	410 (12)	867 (29)	
Myocardial infarction	Yes	982 (10)	578 (60)	186 (19)	218 (21)	**
	No	8185 (90)	4809 (61)	1290 (14)	2086 (25)	
Angina pectoris/CHD	Yes	875 (9)	534 (64)	142 (15)	199 (21)	*
	No	8252 (91)	4836 (61)	1323 (14)	2093 (25)	
Congestive heart failure	Yes	594 (6)	338 (60)	128 (21)	128 (19)	**
	No	8563 (94)	5047 (61)	1343 (14)	2173 (25)	
Other heart condition, eg valve/rhythm	Yes	2264 (22)	1366 (62)	372 (15)	526 (23)	*
	No	6902 (78)	4021 (60)	1103 (14)	1778 (26)	
Stroke/brain hemorrhage	Yes	909 (9)	546 (62)	161 (17)	202 (21)	*
	No	8271 (91)	4849 (61)	1317 (14)	2105 (25)	
High cholesterol	Yes	6117 (65)	3689 (63)	1000 (14)	1428 (23)	***
	No	3033 (35)	1694 (58)	472 (14)	867 (29)	
Cancer (non-skin)	Yes	1940 (20)	1202 (65)	304 (14)	434 (22)	***
	No	7235 (80)	4191 (60)	1172 (14)	1872 (26)	
Alzheimers/dementia	Yes	395 (3)	230 (61)	75 (17)	90 (23)	
	No	8788 (97)	5168 (61)	1403 (14)	2217 (25)	
Depression	Yes	1919 (21)	1211 (65)	309 (14)	399 (21)	***
	No	7246 (79)	4177 (60)	1165 (14)	1904 (26)	
Osteoporosis/soft bones	Yes	1879 (19)	1154 (62)	294 (15)	431 (23)	
	No	7262 (81)	4223 (61)	1172 (14)	1867 (25)	
Broken hip	Yes	355 (3)	205 (60)	58 (13)	92 (26)	
	No	8823 (97)	5190 (61)	1419 (14)	2214 (25)	
Emphysema/asthma/COPD	Yes	1657 (17)	1036 (65)	276 (15)	345 (21)	***
	No	7516 (83)	4357 (60)	1200 (14)	1959 (26)	
Diabetes/high blood sugar	Yes	2808 (30)	1789 (66)	441 (14)	578 (21)	***

	No	6359 (70)	3603 (59)	1031 (14)	1725 (27)	
Any arthritis	Yes	2790 (64)	1681 (62)	494 (17)	615 (21)	**
	No	1464 (36)	856 (62)	238 (14)	370 (24)	
Any heart condition	Yes	3253 (32)	1945 (62)	550 (16)	758 (22)	***
	No	5901 (68)	3436 (60)	924 (14)	1541 (26)	
Any osteoporosis/broken hip	Yes	2085 (20)	1272 (62)	323 (15)	490 (24)	
	No	7056 (80)	4106 (61)	1143 (14)	1807 (25)	
Ever smoke cigarette/cigar/pipe	Yes	5148 (56)	3024 (61)	843 (14)	1281 (25)	
	No	4034 (44)	2371 (61)	635 (14)	1028 (25)	
Currently smoke cigarette/cigar/pipe	Yes	688 (16)	381 (57)	132 (16)	175 (27)	
	No	4456 (84)	2641 (61)	710 (14)	1105 (25)	
Ever used e-cigarette	Yes	484 (6)	284 (59)	78 (15)	122 (26)	
	No	8687 (94)	5109 (61)	1397 (14)	2181 (25)	
Smoke e-cigarette now	Yes	63 (15)	38 (56)	12 (18)	13 (26)	
	No	421 (85)	246 (60)	66 (14)	109 (26)	

[†]Sig indicates significant level according to P values from weighted chi-squared statistics: * for $p \leq 0.05$, ** for $p \leq 0.01$, *** for $p \leq 0.001$; two categories of missing ("don't know", "refused" or "inapplicable/missing") and not missing ("yes" or "no") were tested.

Table S3. Variables for predicting telehealth coverage and Internet access from Random Forest analyses (full version of Table 2).

	Coverage of telehealth				Access to the Internet				
Variable	Est	SE	P value	OR[§]	Est	SE	P value	OR[§]	Sig[‡]
Age	0.10	0.02	0.000	1.65	0.96	0.10	0.000	2.74	***+++
Gender	-0.01	0.00	0.950	0.98	-0.04	0.01	1.000	1.23	
Race/ethnicity	1.14	0.27	0.000	1.92	2.05	0.44	0.000	3.85	***+++
Metro residence	2.00	0.24	0.000	2.00	-0.04	0.03	0.878	1.70	***
Region	-0.04	0.03	0.950	0.92	0.04	0.03	0.072	0.95	
Income	0.10	0.05	0.026	0.49	3.83	0.49	0.000	0.14	*+++
Non-English	-0.02	0.05	0.659	0.67	0.19	0.17	0.133	0.39	
Medicare-Medicaid dual eligibility	0.50	0.53	0.172	0.50	9.78	1.16	0.000	0.13	+++
Medicare Advantage	0.04	0.09	0.320	0.68	0.15	0.10	0.067	1.46	
Part D plan	-0.01	0.00	0.874	0.93	0.00	0.01	0.500	0.63	
Positive COVID-19 test	0.25	0.10	0.005	1.31	0.03	0.14	0.411	0.82	**
Positive COVID-19 antibody test	0.17	0.16	0.139	0.94	0.13	0.08	0.067	1.32	
Able to pay rent/mortgage	0.47	0.21	0.011	1.39	0.24	0.26	0.172	2.13	*
Able to get food	0.99	0.30	0.001	1.58	0.83	0.24	0.000	1.18	**+++
Able to get home supplies	0.30	0.27	0.130	0.95	0.21	0.25	0.195	1.02	
Feel financially secure	0.10	0.16	0.267	1.62	-0.01	0.11	0.520	2.47	
Feel stressed	0.05	0.19	0.398	1.26	-0.05	0.12	0.652	1.12	
Feel lonely or sad	0.38	0.13	0.002	1.05	0.14	0.07	0.018	1.06	**+
Feel socially connected	0.04	0.20	0.415	0.87	0.02	0.09	0.397	0.69	
Weak immune system due to treatment/drug	0.02	0.13	0.453	1.13	0.10	0.15	0.258	0.95	
Weak immune system due to health cond	-0.06	0.01	1.000	1.34	0.01	0.01	0.039	0.98	+
Weak immune system (any reason)	-0.07	0.01	1.000	1.34	-0.02	0.01	0.991	0.99	
Hypertension/high BP	-0.02	0.01	0.986	0.80	-0.02	0.01	0.994	0.60	
Myocardial infarction	0.01	0.09	0.470	0.73	0.02	0.04	0.337	0.68	
Angina pectoris/CHD	-0.01	0.02	0.724	0.98	0.13	0.02	0.000	1.16	+++
Congestive heart failure	0.18	0.23	0.220	0.65	0.15	0.14	0.135	0.66	
Other heart conditions, eg valve/rhythm	0.01	0.01	0.026	0.96	0.04	0.01	0.000	1.02	*+++
Stroke/brain hemorrhage	-0.01	0.17	0.513	0.85	-0.08	0.08	0.837	0.53	
High cholesterol	0.12	0.01	0.000	1.04	-0.02	0.01	0.999	0.95	***
Cancer (non-skin)	-0.04	0.01	1.000	1.12	-0.04	0.01	1.000	0.97	
Alzheimers/dementia	0.06	0.26	0.408	0.85	0.17	0.44	0.350	0.27	
Depression	0.05	0.01	0.000	1.08	-0.05	0.01	1.000	0.88	***
Osteoporosis/soft bones	-0.03	0.01	1.000	0.96	-0.02	0.01	0.987	0.91	
Broken hip	0.17	0.12	0.081	1.06	0.17	0.24	0.235	0.50	
Emphysema/asthma/COPD	0.01	0.02	0.263	1.02	-0.03	0.03	0.820	0.80	
Diabetes/high blood sugar	-0.10	0.01	1.000	1.16	-0.12	0.02	1.000	0.76	
Any arthritis	0.06	0.01	0.000	0.83	-0.04	0.01	1.000	0.82	***
Any heart condition	0.01	0.01	0.141	0.89	0.05	0.01	0.000	0.90	+++

Any osteoporosis/broken hip	-0.02	0.01	0.999	0.99	-0.01	0.01	0.968	0.83	
Ever smoke cigarette/cigar/pipe	-0.04	0.01	1.000	0.96	-0.01	0.01	0.812	1.24	
Currently smoke cigarette/cigar/pipe	0.14	0.15	0.171	0.80	0.07	0.09	0.240	0.61	
Ever used e-cigarette	0.15	0.07	0.016	0.92	0.15	0.06	0.008	1.32	*++
Smoke e-cigarette now	0.61	0.09	0.000	0.72	0.04	0.03	0.152	1.60	***
Interview date	0.21	0.33	0.262	-	0.26	0.22	0.109	-	

Est and SE indicate estimation and standard error for Random Forest variable importance (VIMP).

§OR indicates the survey-weighted odds ratio indicating the direction of effects: if the value is larger than one, the first category of the variable in Table 1 is more likely with a positive outcome than the second category. For example, the odds ratio of age is 1.65, indicating that the 65 to 74 age group was more likely with telehealth coverage than the over-74 age group.

‡Sig indicates significant level according to P values of VIMP: when the outcome is coverage of telehealth, * for $p \leq 0.05$, ** for $p \leq 0.01$, *** for $p \leq 0.001$; when the outcome is access to the Internet, + for $p \leq 0.05$, ++ for $p \leq 0.01$, and +++ for $p \leq 0.001$.

Table S4. Informative variables for predicting telehealth coverage from Random Forest analyses when adding Internet access as an additional predictor.

Variable	Est	SE	P value	OR§	Sig‡
Access to Internet	3.79	0.52	0.000	3.29	***
Race/ethnicity group	1.73	0.47	0.000	1.92	***
Metro residence	2.03	0.22	0.000	2.00	***
Region	0.36	0.05	0.000	0.92	***
Medicare-Medicaid dual eligibility	1.24	0.63	0.023	0.50	*
Positive COVID-19 test	0.42	0.18	0.011	1.31	*
Able to pay rent/mortgage	0.59	0.15	0.000	1.39	***
Able to get food	1.20	0.33	0.000	1.58	***
Feel lonely or sad	0.95	0.23	0.000	1.05	***
Depression	0.04	0.02	0.011	1.08	*
Any arthritis	0.06	0.02	0.001	0.83	**
Smoke e-cigarette now	0.95	0.22	0.000	0.72	***

Est and SE indicate estimation and standard error for Random Forest variable importance (VIMP).

§OR indicates the survey-weighted odds ratio indicating the direction of effects: if the value is larger than one, the first category of the variable in Table 1 is more likely with a positive outcome than the second category. For example, the odds ratio of age is 1.65, indicating that the 65 to 74 age group was more likely with telehealth coverage than the over-74 age group.

‡Sig indicates significant level according to P values of VIMP: * for $p \leq 0.05$, ** for $p \leq 0.01$, *** for $p \leq 0.001$.

2 Figures

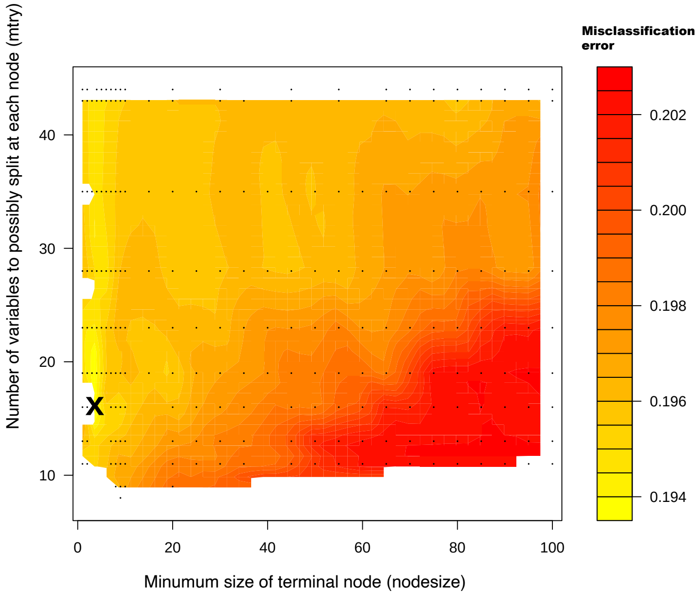

Fig S1. Parameter tuning result from the Random Forest model. Letter X marks the optimal parameters.

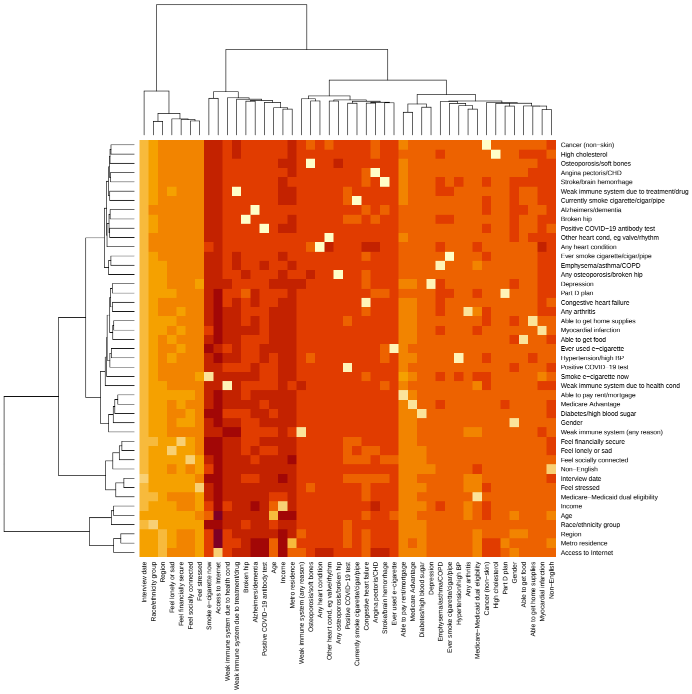

Fig S2. Heatmap of the features utilizing Random Forest maximal subtree analysis. Lighter color reflects a smaller value of minimal depth of a variable in the maximal subtree for the other corresponding variable, indicating a stronger interaction effect.

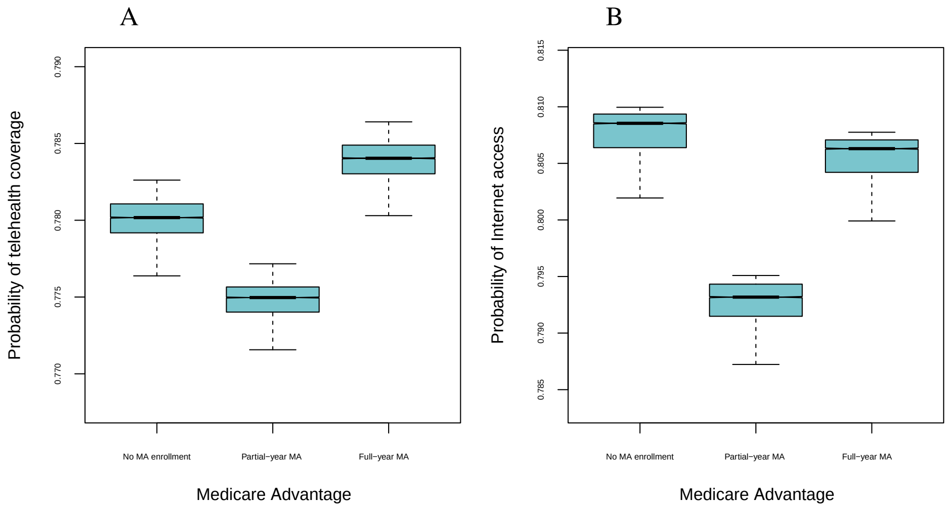

Fig S3. Random Forest estimated probabilities of outcomes plotted against Medicare Advantage (MA). (A) The association between MA and telehealth coverage. **(B)** The association between MA and Internet access.

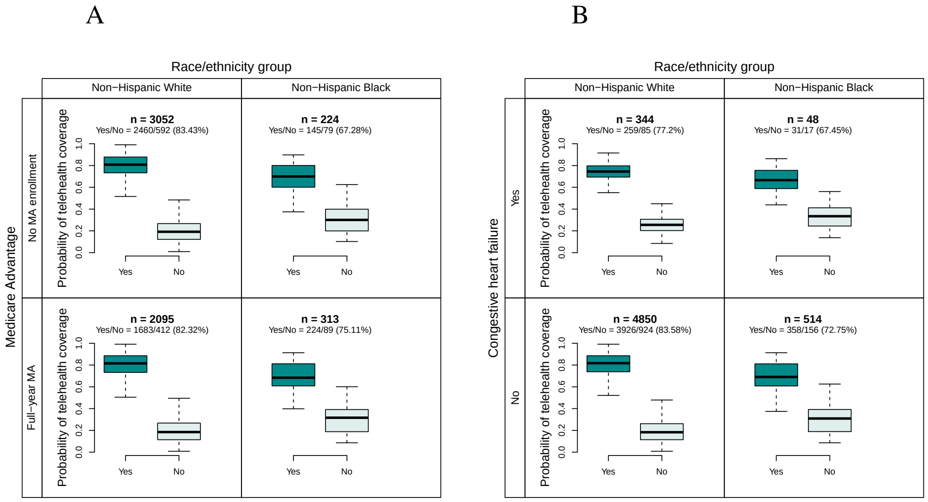

Fig S4. Interactions of variables for predicting the probability of telehealth coverage. The survey-weighted proportions of positive outcomes are listed in the parentheses. **(A)** The interaction between race/ethnicity and Medicare advantage. **(B)** The interaction between race/ethnicity and congestive heart failure (ever).
